# Needle exchange programmes in Visegrad countries: a comparative case study of structural factors in effective service delivery

**DOI:** 10.1186/s12954-019-0323-5

**Published:** 2019-09-03

**Authors:** Iga Kender-Jeziorska

**Affiliations:** Corvinus University of Budapest, Budapest, Hungary

**Keywords:** Harm reduction, Drug policy, Service delivery, Barriers, Challenges, Needle exchange, East-Central Europe (ECE)

## Abstract

**Background:**

Harm reduction services, despite their proved effectiveness in the prevention of infectious diseases, are still underdeveloped in several European states. The situation in the Visegrad Group countries is especially interesting. Notwithstanding the shared history, culture and political situation in the last decades, there are significant differences in the state of harm reduction between the countries.

**Methods:**

The research applies the ecological systems model to identify the structural barriers and facilitators affecting organisations providing needle exchange services. It uses a comparative multiple case study design with embedded units of analysis complemented by within-case analysis to establish the relationship between the number and scope of identified factors and the performance of needle exchange services. The qualitative data were collected through semi-structured interviews with professionals working in needle exchange services in the Czech Republic, Poland, Slovakia and Hungary. Additionally, relevant documents, reports and online sources were analysed.

**Results:**

A total of 24 themes (structural factors) were identified across 11 categories on 3 levels (mesosystem, exosystem, macrosystem). The list includes themes related to the broader society, politics and policy on state and local level, frameworks and amounts of funding, the situation on the education labour market, and attitudes of local communities, among others. The data shows that in the Czech Republic, many facilitators can be identified. In the three remaining countries, on the contrary, one can observe mostly barriers in NSP services delivery.

**Conclusions:**

The study addresses a highly unexplored topic of the functioning of harm reduction organisations in East-Central Europe. It sheds light on the environment of analysed services, identifying a number of structural factors in effective service delivery in the Czech Republic, Poland, Slovakia and Hungary. The research confirms the significant role of the barriers and facilitators in the services’ performance. It highlights the relationships between various elements of the needle exchange programmes’ environment, suggesting holistic strategies for addressing them. It also provides a potential starting point for further research.

**Electronic supplementary material:**

The online version of this article (10.1186/s12954-019-0323-5) contains supplementary material, which is available to authorized users.

## Background

Over the last three decades, harm reduction services have been created all over the world and, especially in Western European countries, have become a well-established pillar of drug policy. There is a substantive body of evidence demonstrating both its effectiveness and efficiency [1]. NPSs, if appropriately implemented in terms of their scope and quality, are proved to prevent the spread of infectious diseases. Hence, policy efforts should now focus on their development. Nowadays, the sound position of harm reduction in Europe is most prominently reflected by the current position of the European Union. The EU Drug Strategy for 2013–2020 is the first-ever strategic document on this level, calling for scaling-up harm reduction interventions and improve access to them as an objective of EU’s drug policy [2].

However, if we take a look at East-Central Europe, a somewhat different picture emerges. According to the Global State of Harm Reduction 2018 report, harm reduction services in Eurasia are significantly less available than in Western Europe. For example, drug consumption rooms are not available in a single country in the Eurasian region [3], contrary to the WE, where 89 such facilities are available in nine countries [3]. Moreover, in the Eastern part of Europe, there are some countries where we can talk about the crisis of harm reduction, understood in terms of declining funding and political support. Opioid substitution treatment (OST) provision is stable in the region, but its coverage is extremely low. Needle exchange programmes’ operation is also restricted, including the recent closure of some/all facilities in countries like Hungary or Bulgaria [3].

The overall poor accessibility and, to a lesser extent, quality of harm reduction programmes in ECE are corroborated by the perceptions of the professionals working in the field. For example, the perceived availability of OST is seen as relatively high in Western (7.39/10) and Southern (7.34) European countries, while it is seen as significantly lower in East-Central Europe (5.49) and Western Balkans (5.27). NSPs are seen as somewhat accessible in WE (6.86) and only moderately available in Southern Europe (5.36) and ECE (5.03) [4].

Due to significant variation within the region, the case of East-Central Europe is especially compelling. Even more so if we take into consideration needle exchange programmes in four members of the Visegrad Group (V4): Czech Republic, Poland, Slovakia in Hungary, as countries of highly similar characteristics and history. According to the cited report of the Civil Society Forum on Drugs (CSFD), the Czech Republic is an outlier, with drastically higher than in other countries perceived accessibility and significantly higher perceived quality of NSPs (Table 1). On the other hand, Hungary’s results are significantly lower in both aspects ([4], pp. 27–28):

**Table 1:** The perceived accessibility and quality of needle exchange programmes in Visegrad countries, the number of needles distributed per client, the geographical coverage of NSPs and the prevalence of HCV among PWID

	Perceived NSP accessibility	Perceived NSP quality	Needles distributed per client per year in 2017^a^	The proportion of cities where NPS are present in 2017^b^	Prevalence of HCV among PWID^c^
CZ	8.9	9.3	199	65%	14.7% (2017)
PL	4.7	7	35	7%	57.9% (2017)
SK	3.5	7.5	184	16%	42.3% (2017)
HU	1.8	5.1	65	21%	49.7% (2015)

Sources [5–7] own data

^a^The calculations of the number of needles distributed per client are based on the data in countries’ annual reports (2017) to EMCDDA, with the exception of: the number of needles distributed per client in Slovakia, where EMCDDA data is not available, and the information on the number of clients were obtained by the author directly from the services

^b^The calculations of the proportion of the cities where NSPs are available are based on the information retrieved from NSPs’ websites and annual reports and the total number of cities in each country

^c^The data are based on countries’ annual reports to EMCDDA

This outstanding performance of the Czech Republic and poor performance of Hungary is further confirmed by some other services-specific impact indicators summarised in the table above (although, according to these indicators, Hungary is not the worst performer).

The differences presented above are quite striking, given the abovementioned high level of similarity between the V4 countries. This similarity, to a large extent, has long historical roots in the peripheral status of East-Central Europe as compared to Western Europe. This character is related to general weakness and instability of nation-states, and their subordination to core states [8] as well as general backwardness in terms of economy, technological development but also political culture and institutions [9]. More recently, the experience of real socialism and Soviet influences had a significant impact on the ECE states and societies. It is argued that this experience caused “civilizational incompetence” [10], resulting in alienation, polarisation and lack of social trust, lack of tolerance, and atmosphere of competition [10]. Concerning more contemporary issues, the similarities lie in rapid political and economic transition after 1989 and participation in the 2004 European Union enlargement. With respect to governance, poorly functioning policy-making processes are of concern in the region. For example, policy implementation is considered a missing link in the region [11]—something crucial in the context of this study.

Another main area of similarity relevant for this inquiry is concerned with non-governmental organisations as the primary (or sole) providers of needle exchange services in examined countries and their involvement in policy-making. It is argued that in ECE NGOs often operate in a vague and/or inconsistent policy environment [12, 13], they experience chronic underfunding [14] and are shoved out to play only a marginal role in governance processes [12].

Given that harm reduction is based on humanistic values of tolerance and respect, and delivered by civil society organisations, one would expect somewhat similar (similarly low) levels of NSPs performance across countries in question.

The final set of similarities is specific to the drug policy area. First, the East-Central European markets, including the drug market, were opened as a result of the fall of the Iron Curtain. The subsequent deterioration of the economic conditions and the level of life [15] resulted in a significant increase of the drug demand and high-risk drug use in the region in the first half of the 1990s [16]. Nowadays, in all four countries, one can see a high prevalence of the injecting use of stimulants; in the Czech Republic and Slovakia, it is methamphetamine [17, 18] and in Hungary and Poland, stimulant-type new psychoactive substances [6, 19]. In all countries, needle exchange programmes are operated mainly (or only) by non-governmental organisations relying only or almost only on the state funds which they typically acquire via public tender procedures.

On the other hand, however, if we consider the legal regulations on psychoactive substances, those in Poland, Slovakia, and Hungary are largely different from those in the Czech Republic. Namely, the latter country decriminalised the possession of illicit substances for personal use nearly a decade ago [17].

Meanwhile, in Poland, drug possession for personal use was criminalised (with the sanction of up to 3 years of imprisonment) by the Act on Counteracting Drug Addiction of 2001 (Article 62) amended in 2005 [20]. The next amendment of 2011 introduced Article 62a, which gives the possibility of criminal proceedings remission given the meeting of a range of conditions [21].

In Slovakia, simple possession is criminalised as well and punish up to 3 or up to 5 years of incarceration, depending on the amount of possessed substance [18].

In Hungary, the modifications of the drug-related legislature have been persistent, with the Penal Code being changed after every change of the government. The last amendment of 2013 reintroduced criminalisation of drug consumption (possession for personal use have always constituted a criminal offence in post-transition Hungary) which is punishable by up to 2 years of imprisonment. At the same time, the alternatives to criminal sanctions were significantly limited [22]. Penalties for the possession of controlled substances vary depending on the circumstances and drug quantity, with up to 2 years of incarceration in case of minor quantities and even 5–15 years for large quantities [19].

### The ecological framework

Over several last decades, there was a shift in thinking about human behaviour (including health-related issues) and its determinants. The focus was relocated to include, besides an individual, broader social context. An increasing number of researches employed an ecological perspective on health [23] and social issues. Ecological perspectives assume that an individual’s behaviour is affected by multiple interrelated factors on various levels, and events occurring at various levels potentially affect any other level. For example, the framework developed, in the context of human development, by Bronfenbrenner [24] differentiates between four levels of interactions/influence: microsystem, mesosystem, exosystem and macrosystem. The microsystem and the mesosystem are characterised by the active participation of an individual in interactions in a single setting (e.g. family) or multiple settings (e.g. interrelations between peer group and school), respectively. The exosystem includes settings interrelated with micro- and mesosystem in a way that they affect one another; however, an individual is not an active actor here (e.g. parent’s workplace). Finally, the macrosystem is concerned with regularities on the level of culture (e.g. beliefs, ideology) [24].

In the area of public health and the “risk environment” and its importance in the context of HIV infections spreading among people who inject drugs was described already two decades ago. It highlights factors like migration, methods of production and distribution of drugs, social norms and culture, as well as policy and legislature [25]. The more recent account on the risk environment (in the context of political transition) differentiates between micro- and macro-level elements of risk environment across four categories: physical, social, economic and policy [26].

Concerning interventions within a health policy area, Bronfenbrenner’s framework was adopted to develop an ecological framework for health promotion. Within this model, five levels influencing individuals’ health behaviour are discussed. Intrapersonal factors include, for example, attitudes and knowledge. Interpersonal interactions and primary groups refer to close relationships and groups like family or friends. Institutional factors include organised social institutions, e.g. schools or workplaces. Community factors include the networks of individual’s primary groups, interrelations of organisations on a local level and local power structures. Finally, public policy level includes legislature and state policies [27].

In this work, the focus is on structural barriers and facilitators affecting NSP delivery, i.e. meso- and macro-level factors lying outside the organisations providing services. Studies analysing the effectiveness of needle exchange programmes identify numerous relevant structural factors: from controversial status of NSPs to various levels of the international drug control regime (including drug enforcement laws, regulations and policies), to behaviours of police officers [28–30], to stigmatisation and social marginalisation of people who use drugs [31], to a country’s economic context, to gender equality, to living conditions and opportunities [32] and to—finally—features of the services themselves [33, 34].

The vast majority of research on the structural barriers to HIV prevention focus on an individual and interrelations between a person’s environment and their behaviour. There are barely any studies putting NSPs in the centre of attention while analysing their context (for exceptions see [29, 35, 36]).

**Table 2:** The summary of the identified barriers and facilitators in the four analysed countries

Level	Category	Themes	CZ	PL	SK	HU
Macrosystem	Morality	Drug use as a sin	NA	− 1	− 1	− 2
		Addiction as a life choice	− 1	− 1	− 1	− 2
	Criminal law	Legal status of drug possession (decriminalisation–criminalisation)	+ 1	− 2	NA	− 2
	State politics	Engagement (engagement–indifference)	+ 2	− 1	− 1	− 2
		Consensus (consensus–opposing views)	+ 2	− 2	− 2	− 2
		Attitudes (hostility–support)	+ 2	0	NA	− 2
	Policy in general	Competition of drug policy with other policy fields	NA	− 2	NA	− 2
	Drug policy	Competition with other pillars of drug policy (i.e. prevention, treatment)	+ 1	− 2	− 2	− 2
		Coverage of demand reduction services in general	+ 1	− 2	− 2	− 2
		Completeness of the demand reduction system	− 1	− 2	−2	− 2
	The framework of HR service delivery by NGOs	Regulations/policies (reasonable–inadequate)	− 1	− 1	− 1	−1
	Resources	Amount of funds (scarce–ample)	+ 2	− 2	− 2	− 2
		Stability of funds (stability–instability)	+ 1	− 1	− 1	− 2
		Donor-imposed limitations	0	− 2	− 2	− 2
		Time-consuming procedures	− 1	− 2	− 2	− 2
		Embedment of harm reduction in policy documents and public tenders	+ 2	+ 1	+ 1	−2
	Education/labour market	Country-level shortage of professionals (e.g. nurses)	− 1	− 2	− 2	NA
		Low level of recognition/respect for social workers and outreach workers employed in harm reduction services	NA	− 2	− 2	NA
Exosystem	Local politics	Motivation (public good–self-interest)	0	− 2	− 2	− 2
		Attitudes (hostility–support)	0	− 1	− 1	− 1
		Scapegoating	NA	NA	NA	− 2
Mesosystem	Community	Not in my backyard attitudes	0	− 2	− 1	− 2
		Conflicts	NA	− 1	− 1	− 1
		Violence	NA	NA	− 2	NA
	Criminal underworld	Direct contacts with the criminal underworld	NA	0	NA	NA

This paper aims to fill this gap and contribute to the study of policy implementation, specifically, the implementation of needle exchange programmes. Focusing on the Czech Republic, Poland, Slovakia and Hungary, it attempts to determine: (i) what are the structural factors affecting the functioning of NSPs, (ii) how they vary between examined countries, and (iii) how they influence the provision of needle exchange services.

## Methods

This study uses an embedded multiple-case comparative case study design, complemented by within-case analysis. A case is needle exchange programmes in a country, while individual service-provider organisations serve as embedded units of analysis. The geographical scope is four East-Central European countries: the Czech Republic, Poland, Slovakia and Hungary and the temporal range encompasses 5 years prior to the data collection. The data was collected, until reaching the sample saturation, in 2015–2019 through semi-structured interviews with 20 key informants. The participants were selected using a mix of purposive sampling and the snowball method. Key informants occupy mostly managerial positions in NGOs providing needle exchange programmes.

The interviews were conducted face-to-face (in public spaces and interviewee’s workplaces) and via online video chats. Informed consent was obtained from all study participants. The average length of an interview was 88 min (the shortest interviews lasted for 37 min and the longest one for 168 min). An interview protocol was used to facilitate the process (the summarised interview protocol can be found in Additional file 1). The protocol included general questions on the everyday functioning of the organisations: the relationships of NSPs with other state actors and institutions (e.g. health care, law enforcement, other services); funding and relationships with donors; and relationships with clients. The questionnaire was slightly modified over time to address new issues emerging from already conducted interviews. The average duration of one interview was approximately 90 min. Conversations were registered (audio) and transcribed verbatim.

The data collected through interviews were complemented by analysis of relevant documents, reports and online resources, primarily the countries’ criminal codes and acts addressing controlled substances, drug strategies and action plans and reports of the Retoix National Focal Points to the EMCDDA. The analysis involved coding the segments of data, using data-derived codes in the iterative process of de-contextualising and re-contextualising data units. Subsequently, aggregated data for each country were reviewed to identify common themes and detect possible irregularities on a higher level of abstraction. Twenty-four identified coherent themes were organised into 11 categories. Subsequently, borrowing from the consolidated framework for advancing implementation science [37], identified themes were rated based on two aspects: the valence and the strength. In other words, it was assessed whether the influence of a factor has a positive (facilitator—“+”), negative (barrier—“−“), mixed (X) or neutral (0) influence, and to what extent it impacts the NSP implementation (on a scale from “−2” to “+2, where “1” indicates weak while “2” strong influence).

## Results

**Fig. 1:**
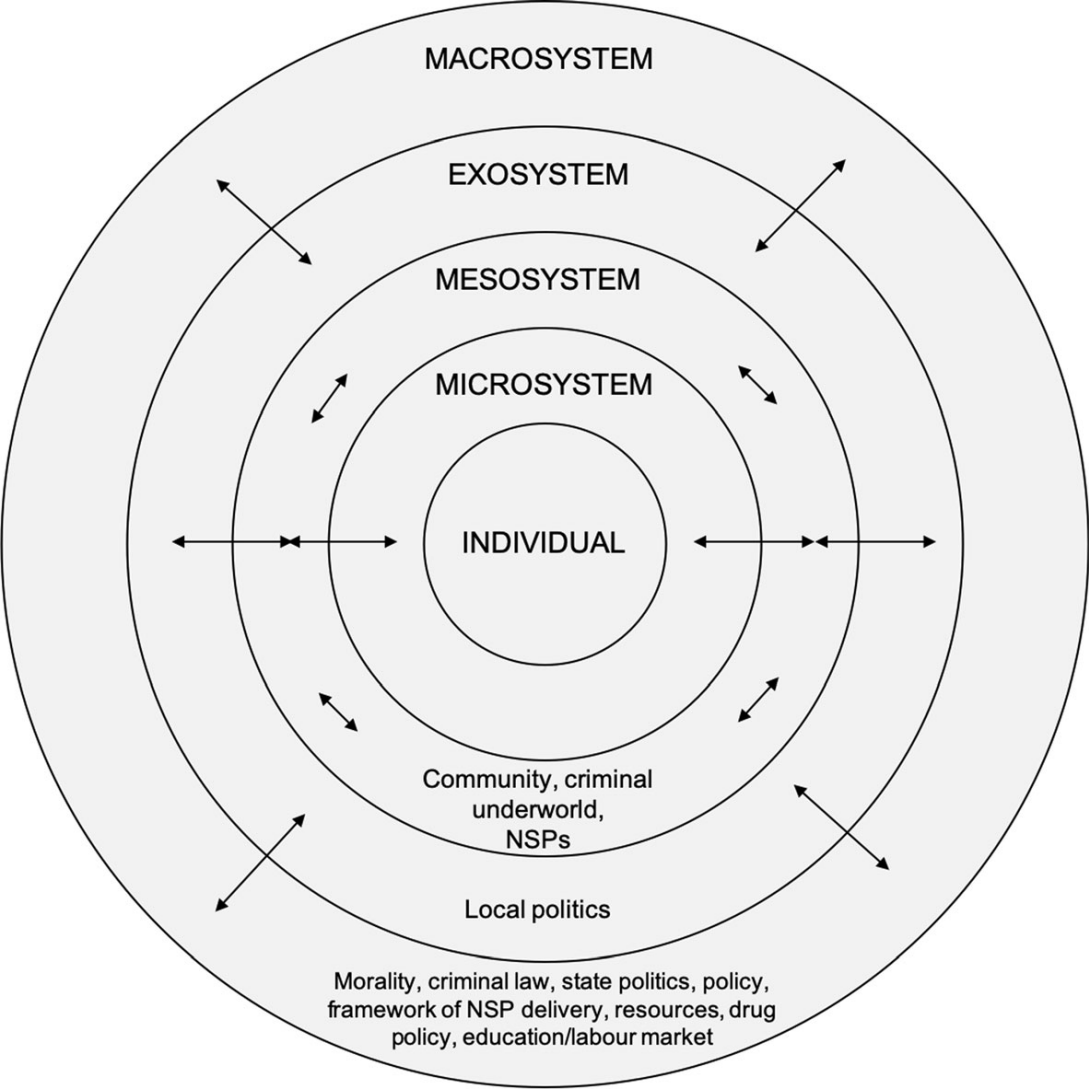
The summary of the categories indicating the location of the identified structural factors within the Bronfenbrenner’s model

Data analysis using the procedures described above allowed for the identification of 11 main categories indicating the location of the existing structural factors on the three levels of the analytical framework. The following figure presents a summary of the categories indicating the location of the identified structural factors within the Bronfenbrenner’s model (Fig. 1).

As mentioned above, included categories and levels are not independent. On the contrary, they interact with one another, often are intertwined and to a large extent affect one another both within and across levels. For example, morality can play a role in determining other themes in the macrosystem but also exo- and mesosystems. At the same time, some of the state policies can affect morality through, for example, enhancing the prevailing attitudes. Furthermore, local community attitudes can influence local politics and further public policies. As a result, differentiating between the categories and classifying the themes turned out to be a challenging task, requiring a reiterative process of re-defining and fine-tuning.

Notwithstanding, 24 themes were identified across all categories. In the following paragraphs, each of them is shortly described. Subsequently, the patterns of barriers and facilitators identified in examined countries are discussed.

Morality category includes two themes: (i) the societal perception of drug use as a sin and (ii) the societal perception of addiction as a life choice (a conscious and informed decision).

Criminal law refers to the legal regulations on psychoactive substances and includes one theme: the legal status of possession and/or use of illicit drugs.

State politics refers to the attitudes of governments, politicians, and other entities involved in the world of politics; it includes following themes: (i) engagement, (ii) consensus, and (iii) attitudes.

Policy, in general, is directly related to politics and agenda-setting and involves only one theme: competition of drug policy with other policy fields.

Drug policy category focuses on the demand reduction system. It includes following themes: (i) competition with other pillars of drug policy (i.e. prevention, treatment), (ii) coverage of demand reduction services in general, and (iii) completeness of the demand reduction system.

The framework of HR service delivery by NGOs refers to the formal arrangements of services delivery as well as attitudes of state actors being responsible for the policy implementation (i.e. “donors”). Themes in this category involve (i) regulations/policies, (ii) red tape.

Resources category captures the features of the funding system and includes following themes: (i) amount of funds, (ii) stability of funds, (iii) donor-imposed limitations, (iv) time-consuming procedures, (v) embedment of harm reduction in policy documents and public tenders.

Education/labour market focuses on the available workforce and includes: (i) country-level shortage of professionals (e.g. nurses) and (ii) low level of recognition/respect for social workers and outreach workers employed in harm reduction organisations.

Community refers to the communities of local inhabitants in the areas of NSPs operation and involves the following themes: (i) not in my backyard attitudes, (ii) conflicts, and (iii) violence.

Local politics focuses on the attitudes and actions of local politicians. The themes include (i) motivation, (ii) attitudes, (iii) scapegoating.

Criminal underworld takes into consideration the characteristics of needle exchange programmes, namely, working in the areas where the criminal activity takes place. The theme identified in this category is direct contacts with the criminal underworld.

The above list, therefore, includes 24 themes—structural factors affecting the service delivery. Here again, the themes are neither mutually exclusive nor independent. Instead, their boundaries are often blurred. For example, the donors’ lack of understanding of low-threshold services can play a role in the adoption of strict reporting policies.

In the following sections, the patterns of identified factors in each of the examined countries and specific ways in which they work are described.

The following table presents the summary of identified structural factors in four examined countries (values are provided for the themes identified as either barriers or facilitators in selected cases) (Table 2).

### The Czech Republic

In Czech society, there is a prevalent opinion that addiction is a blameful life choice, and PWUD are themselves responsible for their situation (KI-15). This attitude towards dependence and—more generally—substance use, does not seem to be however reflected in the country’s legal regulations, where possession of illicit substances for personal use is currently decriminalised and constitutes an administrative offence [38]. Importantly, the abovementioned attitudes are strongly demonstrated among police but especially medical professions (KI–12). People who use drugs are notoriously stigmatised in health services. Although in the Czech Republic, every citizen, regardless of their employment status, is eligible to free public health care, PWUD are sometimes denied services due to stigma and prejudices towards them. Social workers from NSPs often accompany the clients during their visits to health care institutions (KI–14). Since such visits can be time-consuming, the necessity of such interventions negatively affects the capacity of organisations, thus decreasing their accessibility to other clients.

Decriminalisation of drug possession for personal use might have contributed to the facilitation of NSP provision understood as the proportion of the PWID population covered by the services. A significant increase of coverage (almost seven percentage points) took place between 2012 and 2013, and in the following years, the coverage remained at high, approximately 73% level [5]. Regarding the continuity of the NSPs’ relationships with clients, decriminalisation may facilitate it to some extent; however, it seems that many NSP clients are imprisoned for different offences, e.g. drug manufacturing or offences against property committed (KI–13). However, it seems that organisations have the capacity to maintain relationships with incarcerated clients via correspondence or face-to-face meetings.

The police, on its management level, engages in politics by actively opposing the development of harm reductions services, especially establishing first drug consumption rooms and scaling-up the opioid substitution treatment (KI–13). Since there are still no DCRs in the Czech Republic, and the number of OSTs is stable, it is clear that these efforts are successful. Although police’s actions do not aim to impede the functioning of already existing services, they constitute a barrier in service delivery in the context of creating holistic, comprehensive public health responses.

Overall, however, it seems that on the state level, there is political support for harm reduction, and politicians treat it as an essential element of drug policy. It is also confirmed by the public expenditure: 14% of the total drug policy budget was devoted to harm reduction—more than to treatment and prevention together [5]. Outstanding investments in research assessing the impact of various solutions [39], as well as continuity and coherence of Czech drug policy, suggest that politicians are genuinely engaged in this policy field, with the prime minister visiting some of the services in person (KI–13).

The key-informants did not raise competition with other policy fields. Given that public expenditure on drug policy equals to almost 67 million Euro per year, i.e. 0.03% of the GDP [17], it is justified to assume this barrier is not applicable.

On the drug policy level, interviewed experts reported some level of competition for funds between various pillars. Some level of distrust and feeling of injustice can be observed, which is mainly due to perceived unfair funds distribution, providing too much support for less effective and very much cost-inefficient services, for example, based on a therapeutic community model. However, as mentioned above, harm reduction in the Czech Republic enjoys broad support, both financial and political. The analysis of annual reports of low-threshold services listed on the website of Czech National Monitoring Centre for Drugs and Addiction [40] shows that in 2017, the average budget per organisation per year equalled to 381,006 Euro,^1^ which translates to 243 Euro per year per client. Moreover, from 2008 through 2017, the total budget of analysed NSPs increased by 158.8%. As such, this theme is considered not applicable in this case.

Although the demand reduction system does not appear among identified factors, it deserves a moment of attention. Interviewed experts have expressed some concerns about insufficient services coverage in terms of reaching the target population and waiting time to enter the treatment. However, the data from the interviews and official reports show that low-threshold NSPs cover approximately 70% of the people who inject drugs [5]. Further, in 2017, 41,000 individuals were undertaking treatment and 5000 individuals received OST [17]—86% and 81% of the estimated number of high-risk drug users and non-buprenorphine opioid users, respectively. Key informants’ critique seems to be influenced by their context, i.e. the relatively good overall situation of the field. One can also argue that any coverage below 100% is insufficient. Nevertheless, the Czech performance, in this case, is outstanding, especially in the context of the region. For that reason, this theme is considered not applicable.

What indeed considered problematic in the Czech Republic is the fragmentation of the social care system. Some services, e.g. shelters, subsidised housing or protected workplaces for people who use drugs are not in place (KI–13). Moreover, the cooperation between various services, e.g. NSPs and hospitals or NSPs and treatment facilities, is not institutionalised. Although the system offers a range of services, cooperation between them takes place on an individual (as opposed to organisational) level and case-by-case basis (KI–14). In practice, this means that each time a client of NSP wishes to enter the treatment or use any other facility, the entire procedure of contacting entities one-by-one and asking about possibilities. This, of course, is a time-consuming activity and as such negatively affects the capacity (and, in consequence, effectiveness) of NSPs. However, numerous NPSs in the Czech Republic are established within bigger organisations offering various other interventions. Therefore, in many cases, there is a possibility to refer clients from one service to another within one organisation (e.g. from NSP to OST or abstinence-based treatment).

The framework of services delivery by NGOs has one major drawback; it does not differentiate between various types of social services in terms of the care-related requirements [41]. As a result, NSPs fall into one category with facilities providing inpatient care for elderly or orphanages. The quality standards for social services require all social services to develop an individual plan of work with each client visiting [42]. This misunderstanding regarding the characteristics of low-threshold NSPs (where often clients spend in the facility only a couple of minutes to exchange the equipment) results in unnecessary administrative burden for the employees of NSPs who need to comply with the regulations. Again, this negatively affects the programmes’ effectiveness by decreasing their capacity.

The financing system in the Czech Republic is multi-source, based on tenders and with each donor having their limitations regarding what the money can be spent on (e.g. salaries, injecting paraphernalia). These limitations combined with short-term project tenders and lengthy grant proposal acceptance procedures (which result in the scarcity of resources in certain months of the year) result in need of extensive planning throughout the year and necessity of writing project proposals frequently. However, as mentioned above, the majority of NSPs in the Czech Republic are parts of bigger organisations which have own financial-administrative departments. As a result, the need for extensive planning only partly affects the services directly as the majority of work is done on the organisations’ central level. Importantly, the Czech action plan on drugs for 2016–2018 takes the possibility of implementing multiannual funding schemes and unified project submission, including multiple donors under consideration [43]. The amount of funding for NSPs, as demonstrated above, is high. Although theoretically, the sustainability of financing is uncertain, interviewed experts see funding as stable, which allows them to plan for the future.

The country is experiencing a considerable shortage of labour force in medical professions [44]. As a result, organisations struggle to find nurse and addictology doctor employees. Hence, the labour market situation prevents NSPs from improving the quality of their services.

Nowadays, local communities are perceived to be nationalistic, xenophobic, and generally less accepting (KI–13). People who use drugs are a convenient enemy who fits people’s more general attitudes. However, after many years of community work and education, the conflicts between NSPs and local communities are hardly present (KI–12). Overall, the attitudes of local communities may affect the PWUD, but they do not have any influence on services’ operation.

The political situation on the local level is somewhat differential and dependent on people holding positions in local authorities at given moment. In some cases, local politicians seem to use drug policy topic for their political goals. In others, they are perceived to be highly engaged and motivated to find working solutions. Possible instrumental use of drug topic does not affect NSPs operation, however. Experts report that in general, the attitudes of local politicians are neutral towards harm reduction services. In some cases, advocacy and educational work are necessary among representatives of local authorities (KI–15).

### Poland

According to the interviewed experts, in Polish society drug use is perceived as sin, crime. Substance dependence is seen as a conscious choice of a lifestyle. These widely shared societal attitudes are reflected by the attitudes towards drugs in general population surveys. Although drug consumption is not criminalised in Poland, almost 80% of Poles think cannabis consumption should be prohibited, and 90% that heroin consumption should be illegal [45]. Marginalising attitudes are also reported in medical professions, resulting in a denial of health services for people who use drugs, even in extreme situations. It is reported that NSP staff spends a considerable amount of time accompanying clients in contacts with public institutions (KI–4).

These attitudes are reflected in the legislation. The criminalisation of drug possession (any amount of any illicit substance) results in frequent incarceration of NSP clients, thus interrupting the continuity of relationships with them. Services devote their time to provide legal help for the clients to prevent their incarceration (KI–3). Such legal advice can undoubtedly be considered harm reduction activity, though it rather addresses harms resulting from certain drug policies, not the use of drugs. In the absence of the criminalisation of simple drug possession, services would enjoy more capacity for other tasks. The effectiveness of NSPs, based on trust and long-term relationships, is being undermined by the contradicting mechanisms of law enforcement.

Politicians on the state level are perceived to be indifferent and drug policy as never being a priority in the Polish governments’ agenda. Condemning public opinion on drug use makes this area even more unattractive for decision-makers; being driven by self-interest, they rather do not risk their positions addressing highly controversial policy fields (KI–4). In consequence, they tend to neglect drug policy altogether; drug policy is addressed in the programme of only one political party being currently in the Parliament, and the reference is limited to “fight against NPSs and drug crime” [46]. Occasional ad hoc activity demonstrating firm positions against substance use (e.g. raiding shops selling NSPs, a total ban on NPSs) can be observed, accompanied by morally loaded official statements in the face of some crisis, e.g. talking about “dealers of death” in the context of rising NPS poisonings [47]. This results in an unfavourable environment for harm reduction organisations on a national level and likely affects other structural factors, e.g. funding or attitudes of local communities. Nevertheless, harm reduction specifically does not seem to be within the area of attention of state politicians.

The lack of political interest not only in harm reduction but in drug policy in a broader sense results in the atmosphere of competition with other, more politically attractive policy fields. Interviewed experts tend to believe that even the scarce funds currently allocated to drug policy would be likely transferred to other policy areas (with possible minimal funding retained for abstinence-based treatment and recovery) if no external pressures and expectations (e.g. of the EU) were in place (KI–4). The public expenditure on drug policy in Poland was estimated at 35 million Euro or 0.01% of the GDP [48].

Drug policy in Poland has been strongly focused on law enforcement and based on the firmly rooted abstinence paradigm. Prevention, long-term inpatient recovery services as well as abstinence-based ambulatories enjoy the highest political and social support, although support for harm reduction activities is one of the tasks included in the National Programme on Health [49]. NSPs try to strengthen their relative position through advocacy efforts, yet harm reduction interventions are still marginal.

It needs to be noted that, recently, the situation slightly improved due to resources from so-called gambling fund partly transferred to support public health interventions [50], including drug harm reduction organisations. The level of financing is still very low, however. Although aggregate data on public expenditure on specific pillars of drug policy is not available, the amounts of funding planned for various interventions in public tenders are quite informative. For example, tenders funded by the gambling fund for 2019–2020 devote approximately 190,000 Euro annually for needle exchange programmes [51], while over five times this amount is secured for various prevention activities [52]. The analysis of the data acquired from 3 out of 12 organisations officially operating NSPs shows that in 2018, the average budget per organisations equalled 57,007 Euro and the average budget per client 158 Euro.

Except being generally low, the funding is also somewhat unstable. It relies on short, mostly 1-year-long projects based primarily on tenders [53]. As a result, organisations find it challenging to develop long-term strategic plans. Applying for funding is highly time-consuming due to multiple sources of financing. Moreover, each donor has their own limitations regarding the categories of expenses. The shortage of financial resources and the design of the funding schemes result in the need for extensive planning throughout the budget year to maintain the functioning of services. Even more importantly, it also can occasionally directly impede the effective service delivery as organisations need to restrict the scope and magnitude of their services, e.g. by limiting the number of needles distributed per person per occasion (KI–1).

Demand reduction system is ineffective. The coverage of most of the services is very low, e.g. in 2017, there were 24 detoxication centres, 22 OST programmes and 28 institutions providing HIV testing [6]. Only18% of high-risk opioid users were in substitution treatment [54]. Experts report that waiting time for detoxication is usually several weeks, and for treatment, it reaches even several months (despite the visible domination of this pillar of drug policy). As a result, many of the clients who, at a certain point, are willing to enter such services, ultimately give up. As a result, and in combination with perceived hostile attitudes of various state institutions’ personnel, harm reduction organisations struggle with helping their clients to step forward and ensure the access to the services clients need.

The state actors responsible for implementing the policy and allocation of funds impose strict reporting policies which are inadequate for such type of services and put a great administrative burden on NSPs’ staff (KI–3). Moreover, some of these requirements (e.g. requiring a signature of a client under each intervention) are in clear conflict with the fundamental principles of low-threshold NSP, e.g. the principle of anonymity. As a result, the trust between services and their clients is put at risk. Experts report that a project, including visiting clients in prison, was terminated by the donor due to the lack of possibility of obtaining inmate clients’ signatures confirming the intervention implementation (KI–3).

Elaborated time-consuming explanations are also required in cases where the number of persons using some service does not correspond precisely with the number of persons the service was planned for. Such strict approach suggests a low level of trust to professionals working in the services and lack of flexibility and readiness to take into consideration the specificity of working with PWUD.

Outreach worker profession does not enjoy much respect, especially within the demand reduction system. It seems to be perceived as the first step or a transitionary stage on a professional’s way to become an addiction therapist—a role held in high regard (KI–1), likely due to the prevalent abstinence paradigm. As a result, NSPs often strive to find suitable employees. This, perhaps combined with the scarcity of funds, resulted in the decrease in the number of NSPs by almost a half (from 21 to 12) between 2002 and 2017 [6, 55].

Local communities demonstrate strong “not in my backyard” attitudes, fuelled by the belief that it is the services which attract PWUD to certain areas (contrary to the actual practice of establishing facilities in places where PWUD are already present). Numerous protests have been organised in locations cities where either NSPs or treatment ambulatories have been (planned) to open [56–60]. Such attitudes, moreover, are not only manifested against drug-related services but also interventions for other marginalised groups. Local inhabitants protest against social cooperatives employing socially excluded populations [61], shelters for homeless [62, 63] and psychiatric wards in hospitals [64]. Conflicts between local communities and NSPs are thus not exceptional. In the experts’ opinion, confirmed by the above sources, attempts to discussing the problems often fail due to high levels of fear, prejudice and stigmatisation. Organisations need to actively engage in extensive community work to be able to establish services in the first place and not always successfully.

Local politicians exhibit a variety of attitudes, typically consistent with their parties’ orientation, with conservative ones being especially active against NSPs [65]. Overall, they seem to be primarily motivated by self-interest and keeping their positions, thus maintaining a safe distance from NSPs. As a result, the situations can happen when, being under the pressure of the local community, local politicians favourable to NSPs, do not agree on establishing services in areas of their responsibility [66].

In some cases, organisations deliver their outreach services in zones where selling drugs also takes place. Local dealers exhibit a distrust and hostility towards the outreach workers, resulting in potentially dangerous situations which, if not being dealt with professionally, can result in physical violence (KI–3). Work in such surroundings requires careful and significant engagement in the interactions/relationships with local dealers to provide the organisation’s employees with a relatively safe working environment.

### Slovakia

Thinking about drug use in Slovak society is dominated by its perceptions as a weakness or crime. Slovak society exhibits attitudes supporting criminal law as the most effective way to tackle drug use, and PWUD are often alienated [67, 68]. These cultural-societal factors, to a large extent, determine the paradigm adopted in the drug policy field and influence services’ working environment in numerous ways [69].

Decision-makers on the state level have little interest in the area of drug policy, which has never been a priority issue in Slovakia, likely due to its political unattractiveness (KI–16). For example, at the beginning of the 2010s, a transfer of authority took place, placing the responsibility for drug policy under the Ministry of Health, instead of the Government Council for drug policy, which was in charge of it before [70, 71]. It is argued that the Ministry of Health is characterised by lower political influence and lower capacity [72], which may suggest modest interest in this policy area. Interviewed experts see politicians as being populist and driven by the self-interest of gaining and keeping the power. For that reason, they argue, the plans of drug decriminalisation were abandoned [73]. High fragmentation of the availability of data from the country’s reports to EMCDDA can also suggest low interest in drug policy altogether.

Although drug possession is criminalised in Slovakia, it was not identified as a barrier in service delivery. This may be due to somewhat different focus and methods of work of Slovak NSPs. Contrary to Polish, Czech and Hungarian programmes, which develop long-term relationships with clients and attempt to provide the most comprehensive care possible, Slovak organisations focus mostly on the needle exchange and accompanying social work of modest scope. They normally do not assist clients in contacts with various institutions, and they do not develop individual re-adaptation work plans with clients.

Low-threshold harm reduction programmes are seen as competing for funds and political support with other types of interventions in the field (KI–15). Harm reduction is marginal, while the policy focus is on prevention and treatment. In 2006 (the most up-to-date comprehensive data on public expenditure in Slovakia), the public expenditure on harm reduction was approximately 97,000 Euro, while almost 570,000 Euro was spent on prevention, 380,000 for treatment and social reintegration [74]. In 2017, the Ministry of Health, based on a public tender, supported NSPs with 53,000 Euro—50% less than originally requested by the organisations and nine times less than was provided for prevention and treatment [75].

The demand reduction system is highly incomplete and deficient. OST has very low coverage, with only 620 clients, i.e. approximately 12% of the estimated number of high-risk opioid users were receiving such treatment in 2017 [76, 77]. Interviewer experts report that there is no housing and work support for people who use drugs. Moreover, health insurance is required for receiving Hepatitis C treatment, which makes most of the NSP clients ineligible. At the same time, HCV prevalence among treatment clients in Bratislava was over 40% in 2017 [18].

Interviewed experts notice a crisis of social work in Slovakia. The problem with the adequacy of education has been raised in the scholarly literature as well [78]. It is argued that the high popularity of this profession several years ago resulted in the emergence of colleges offering poor-quality education and issuing numerous diplomas with students’ minimum effort. As a result, societal respect for social work has drastically decreased (KI–16). The current consequence of this process is a shortage of well-qualified personnel willing to work with people who use drugs. On the other hand, legal regulations on social services [79] make it very difficult to hire peer workers who usually have a criminal record (KI–14), the ones who are the most effective in reaching out to hidden populations.

State actors responsible for policy implementation, according to the interviewees, impose extensive reporting requirements. The considerable amount of time that NSPs employees need to devote to project-writing and reporting is at the expense of organisations’ clients (KI–14). Interestingly, one of the strategies adopted by one of the organisations is not taking part in tenders, where donors have requirements perceived as unreasonable (KI–16).

The amount of funds available from the state is perceived as insufficient. The organisations need to fundraise continuously (KI–15). However, the absolute numbers are relatively high in the context of the region. The analysis of organisations’ annual reports published online shows that in 2017, the NSPs funding equalled to approximately 135,000 Euro per organisation [80–82]. Moreover, the total budget of the three organisations currently existing in the country almost doubled over the period 2008–2017. Nevertheless, it does not necessarily demonstrate the improvement of the overall situation. In recent years, several organisations were closed down, allegedly to the lack of funds. Currently, only three NSPs operate in Slovakia.

As can be seen in the organisations’ annual reports, the funding system is multisource and includes mainly regional and local governments, the Ministry of Health and the Ministry of Social Affairs [80–82]. As mentioned above, financing is based on public tenders and 1-year projects [75], which makes the organisations write numerous elaborated applications each year. Such funding framework is uncertain and unstable, but the interviewed experts do not express concerns about the future sustainability of their organisations. However, this can be due to the fact that they are the only NSP providers remaining in the country. Relevant ministries administer available EU Structural Funds. Having control over these resources, the responsible civil servants tend to limit the amount of organisations’ funds allowed initially within a project (KI–14). Informants also raised the problem of corruption in the Ministry of Health [83], which results in favouring certain applicants, e.g. TV channel producing moralising videos about drug use. Indeed, the results of public tender from the website of the Ministry of Health show that in 2017, a media company was granted nearly 50,000 Euro for that purpose—10% of the total expenditure and only 3000 Euro less than all NPSs altogether [75].

The donors’ limitations concerning the categories of expenses and the instability of the funding over a year (due to lengthy tendering procedures) result in need of extensive planning of the entire budget year (KI–14). The rigidity of funding rules does not allow to respond to dynamically changing circumstances and evolving needs of clients (KI–16).

Experts believe that local communities are rather unaware of how harm reduction works. It is widely believed that organisations are helping their clients to use drugs and that the existence of service in central location attracts PWUD (KI–15). This can be related to the marginal role of harm reduction in Slovak drug policy and strong law enforcement and prevention focus. Some local inhabitants actively oppose HR organisations establishing in their areas through organising protests. In the past, the instances of verbal and physical violence towards outreach workers were not exceptional. Nowadays, although conflicts are still present, organisations try to mitigate them. However, the violence has not disappeared completely; it now tends to be directed exclusively towards NSPs’ clients (KI–15). Certainly, this affects the work of the services, whose employees devote time to community work and protecting their clients. Such situations can also discourage PWUD from using services altogether, further hindering the effectiveness of NSPs’ work.

Local politicians are not perceived, as a rule, as hostile towards organisations. They are rather seen as driven by self-interests (gaining and keeping power) and using arising opportunities (e.g. picturing oneself as the defender of the district/area) for political gain (KI–14).

### Hungary

According to interviewed experts, in Hungary, the use of psychoactive substances is still a taboo. Drug use is considered as a weakness, sin, and addiction tends to be perceived as consciously chosen, blameful way of life. It is confirmed by the research of attitudes, which demonstrates that the vast majority of Hungarians do not want any contact with PWUD. Namely, 64% of them would not like to have a drug-dependent person as a neighbour. It is the highest result of all group included in the study (at the same time, the result for people with a criminal record was 50%). The research also show that the public opinion, full of negative stereotypes and driven by moral panic, is detrimental to various services, including harm reduction [84]. This general attitude towards psychoactive substances and people who use drugs is very strongly reflected in the country’s current drug policy. The national anti-drug strategy titled “Clear consciousness, sobriety and fight against drug crime” and includes a message “to those people who have tried drugs: a clear indication that they take a risk by abusing substances, and they can harm themselves and their environment” [85]. It also is noticed in the attitudes of public servants in various state offices, as well as in public health care institutions, where frequently PWUD are denied services (KI–9).

Drug consumption is punishable by up to 2 years of imprisonment [86], and the possibility of diversion is very limited to one every 2 years [86]. Such shape of criminal law favours frequent incarceration of PWUD, thus negatively affecting their relationships with harm reduction services. Besides breaking NSPs’ relationships with clients, such legal environment can also be a deterring factor in services use, especially in the absence of any form of clients’ protection such as, for example, a non-interference agreement between the police and Budapest NSPs which was in place in 2004–2013. The criminalisation of substance use (as opposed to the criminalisation of possession only) also changes the legal environment of the NSPs themselves. Since facilitation of committing a crime is also punishable, services’ employees can also be at risk; it is only a matter of political will whether distributing needles is interpreted as an accessory in a criminal offence.

Interviewed experts are strongly convinced that drug policy is beyond the area of interest of state politicians. Recent research on the topic confirms this observation: in 2010–2018, the word “drug” appeared in parliamentary speeches 608 times, out of which 140 took place in 2013—the year of adopting drug strategy. Furthermore, “harm reduction”, “low-threshold” and “needle exchange” appeared 290 times during the same period [87]. Moreover, it can be argued that this indifference manifests itself in the dynamics of legislation: Hungarian drug policy lacks continuity and strategic thinking; legal regulations on psychoactive substances (de- and re-criminalisation of drug consumption) have been changed each time the ruling party has changed [22].

Politicians on the state level are seen as self-interested and reluctant to show support for harm reduction, which is clearly reflected in the country’s drug strategy (see below). They are thought to be populistic, manipulative and intending to destroy organisations they perceive as hostile or representing contradicting worldviews (KI–11), which is clearly negatively affecting the effectiveness of service delivery.

The National Anti-Drug Strategy for 2013–2020 adopts a strong criminal justice approach and abstinence paradigm. One of its long-term goals is that “shall be drug-free until 2020, in spite of the fact that this may seem unreal, based on the trends in the world and in Hungary” [85]. This goal is clearly in conflict with harm reduction principles and reflects opposing views of the decision-makers. Harm reduction activities are part of the Hungarian Anti-Drug Strategy only as an auxiliary to recovery services, and PWID are pictured as a burden for the society. Such an approach, it is argued, enhances the taboo around drugs and discourages PWUD from using harm reduction services [88].

As already mentioned, drug policy does not keep a high position on Hungarian politicians’ agenda. There is some perceived competition for political and financial support between drug policy and other policy fields, with drug policy remaining marginalised. The most up-to-date data on public expenditure shows that in 2007, 0.04% of Hungarian GDP (39 million Euro) was spent on drug policy. Today, this number is very likely much lower. While expenditure for harm reduction constituted 4% of this amount, altogether 20% was spent on prevention and treatment and 75% on law enforcement [19]. Hence, even before the harm reduction crisis, which began in 2010, this area was not a priority in Hungary.

The demand reduction system is ineffective primarily due to low coverage of existing services (OST, treatment), and lack of other, crucial for clients’ re-adaptation, e.g. housing, protected workplaces. In 2015, nearly 700 individuals were covered by OST [7], which is approximately 20% of the population using opioids. In the same year, there were 86 outpatient and 13 inpatient entities providing treatment, reporting to the National Focal Point [7].

Organisations attempt to establish some relationships, based on personal contacts, to provide their clients with holistic care (e.g. with doctors or treatment centres). Still, due to strong prejudices (e.g. in the health care system), PWUD often have difficulties in accessing various services (KI–8). NSPs’ social workers assist their clients in contacts with various institutions to facilitate them. Such activities, being rather time-consuming, decrease the capacity of organisations to work on other issues, e.g. individual social work with clients. There is also a shortage of professionals willing to work in the field, which makes organisations struggle to find suitable employees.

Donors impose extensive paperwork and reporting on every activity of clients, which is seen as counterproductive and creating an unnecessary administrative burden (KI–9). The analysis of annual reports of NGOs providing NSPs (e.g. [89–91]) shows that financial system is multisource (though virtually all donors are state actors), with every donor announcing various tenders, having different priorities and limitations regarding what the money can be spent on. This results in need of writing numerous project applications every year to ensure the continuation of the services. Importantly, it is reported that “harm reduction” does not appear in any tenders announced. This information is corroborated by Hungary’s report to the EMCDDA, which states that “no public call for tender had been issued since 2012”, yet, contracts signed earlier are renewed each year [7]. Nevertheless, the services need to find solutions to maintain needle exchange services and try to obtain funding via other service categories. Organisations have to plan extensively and carefully, anticipating possible shifts in clients’ needs, especially given the dynamic situation on the NPS market. The aforementioned structural factors cause a further decrease in organisations’ already limited capacity, and thus the effectiveness of service delivery.

The funding is characterised by a high level of instability (1-year-long projects) and unpredictability—organisations can never know whether they will receive the funding or not. The difficult political situation of harm reduction and its inferior place in the drug policy system enhances this uncertainty. Organisations strive to survive; the amount of funds is meagre. The data of two organisations providing information show that the budget per client was only around 65 Euro per client in 2017. The data from the Directorate-General for Social Affairs and Child Protection, responsible for the funding of the needle exchange show that in 2017, 46 low-threshold projects were supported with 140,000 Euro altogether—approximately 3000 Euro per project per year.

The local community is primarily considered as hostile towards PWUD and ignorant, and therefore, easy to manipulate by politicians (KI–7). In general, it seems that the majority of services operate relatively smoothly, and conflicts with the local communities are not emerging. However, the information acquired from the interviewed experts suggests that “not in my backyard” attitudes are present where services are visible (KI–8). Several years ago, serious conflicts with local communities escalated in Budapest, being fuelled by the actions of local politicians.

Although the relations of services with local inhabitants were rather neutral in the past, they had changed when the mayor of the eighth district of Budapest started a scapegoating campaign against an NSP working in the area, accusing it of attracting PWUD and making it responsible for the injecting equipment abandoned on the streets [91]. Numerous slander articles were published on the district’s official website [92], and protests of local inhabitants organised [93]. In the face of the state government’s refusal to provide financial help, the NSP was closed. The closure of this needle exchange was followed by another one, due to the sudden increase in client turnover after the first closure. NSP clients became visible on the streets, which triggered complaints from the local community and the district mayor withdrawing the licence for providing needle exchange soon after. Both closed programmes were responsible for providing approximately half of the country’s needles [94].

### Structural barriers and NSP effectiveness: similarities and differences

As the above within-case analysis demonstrated, significant differences can be observed between examined countries. Especially salient, although not surprising, is the case of the Czech Republic. It seems that the majority of the structural factors identified work as facilitators rather than barriers in NSP service-delivery. On the contrary, in Poland, Slovakia and Hungary, the majority of factors seem to have a detrimental impact on NSPs.

The identified factors are not independent of one another. On the contrary, the ecological model assumes interrelations within and between various levels of the environment. For example, the public opinion on drugs and attitudes towards PWUD possibly affect the legislation and drug policy, which, in turn, affects the local-level policies. Such interactions are demonstrated in the data. In Slovakia, the prevalent belief in law enforcement as the most effective tool in addressing drug use resulted in policy strongly focused on criminal justice and marginalising harm reduction. In Hungary, on the other hand, the current drug strategy arguably shifted the discourse, strengthening the taboo around substance use and deterred PWUD from using the services.

Importantly, in all countries, the interviewed experts highlighted the interplay between local politics and attitudes of local communities. Politicians (both on state and local level) are seen as opinion leaders responsible for shaping people’s views. The examples of the Czech Republic and Hungary show that single factors have a significantly lower impact on the NSP service delivery than the combination of thereof. Namely, in the Czech Republic, despite generally negative attitudes of the local communities towards both PWID and NSPs in the past, intensive community work and generally neutral attitudes of local politicians allowed for achieving a situation where NSPs relations with local inhabitants are satisfactory. On the other hand, the case of Budapest shows that political action was needed to fuel people’s pre-existing fears and prejudices to damage previously acceptable situation in the neighbourhood and trigger open conflict.

Similarly, the majority of the identified barriers and facilitators on the macro level are intertwined. In Poland, Slovakia and Hungary, the low interest in drug policy results in scarce resources. Public condemnation of drug use and support for law enforcement measures and abstinence-based services, in turn, result in low political support for harm reduction and, in consequence, in uneven distribution of the scarce resources within the policy subsystem, with harm reduction being on the margins. In the Czech Republic, in turn, relatively high political interest in the drug policy area and undoubted support for harm reduction interventions result in resources distribution favourable for NSPs.

However, there seems to be a similarity between the countries on the macro level. The data shows that in all analysed countries, substance dependence is considered a life choice, i.e. a result of more or less conscious actions of individuals. It is clear that its impact on NSPs operation varies between countries, however. Research shows that Czech students disagree with cannabis decriminalisation at a similar level to Polish students [95]. Still, the legislation on the matter varies substantially between these two countries. It seems that the fundamental difference is the apparent lack of impact of societal views on the legislation, politics and policy. One scenario is that politicians act against the will of people and take a position of opinion leaders. Another possibility is, however, that these societal views are weaker in the Czech Republic than in other countries. If this is the case, one of the explaining factors can be religion: while the Czech Republic is the most secularised country in Europe, Poland is the most religious one, with Slovakia and Hungary ranked in between [96].

Another interesting issue involves Poland, Slovakia and Hungary, and state politics. In all three countries, the indifference of politicians was observed with regard to the drug policy field. However, in Hungary, the low level of existing interest is characterised by relative hostility towards harm reduction, while in Poland and Slovakia, neglect and abandonment of the area seem to dominate. Results are similar but have different dynamics. Namely, in Poland and Slovakia, the deterioration of the needle exchange situation has been gradual, with 1–2 organisations closing services annually in Poland, and 1 organisations closing in Slovakia recently (importantly, the biggest NSPs in Slovakia are still operating). On the contrary, in Hungary, the change was unexpected and quick—two biggest NSPs in the country were closed down within several months from one another.

Shortage of professionals was reported in the Czech Republic, Poland and Slovakia, yet again, its impact varies. The reason for that is the kind of professional missing from the labour market. In the Czech Republic, it is health professionals, i.e. nurses and addictology doctors. In Poland and Slovakia, in turn, it is social workers (due to the lack of respect for the profession and due to the features of the education market, respectively). Of course, needle exchange can operate smoothly without medical staff, but it cannot be implemented without the basic workforce, hence the difference in the impact.

In sum, in the Czech Republic, the environment of harm reduction services has been stable over the years, with rather high political support, participatory processes in policy-making and favourable legal regulations (decriminalisation of simple possession) introduced in 2010. Needle exchange programmes in the country have been steadily developing, with the number of clients increasing by 53% between 2006 and 2016, the number of services increasing 2.5 times and number of needles distributed in the country skyrocketing by 1130% in 1998–2016 [5].

In Poland and Slovakia, general indifference seems to be dominant: lack of political support, insufficient funds, rather moral (than evidence-based) approach to drug policy. The number of NSPs in Poland fell from 23 to 12 between 2001 and 2015 [97], the total number of needles distributed decreased by more than 3.5 times (from 668,152 to 181,180) in 2002–2013 [98], and the number of clients almost six times, from 7763 in 2001 [99] to 1360 in 2015 [97]. In Slovakia, the number of organisations running NSPs fell from six in 2005 [74] to three in 2018 (own data). The number of clients in two out of three organisations existing today has been stable (11% increase between 2008 and 2017; own data), so has the number of needles distributed in the country: 362055 in 2005 [74] and 357,705 in 2016 [76]. It can be thus concluded that the presence of a significant number of structural barriers, including the lack of political support and sufficient funding, is related to stagnation or deterioration of services’ development over time. Further research is needed to assess the role of individual factors in explaining the dynamics of changes in Poland and Slovakia (deterioration versus stagnation).

The crucial role of structural barriers is especially visible in the case of Hungary. The environment of harm reduction was continuously improving during the 2000s, with significant political support and inclusive processes of policy-making (e.g. involving NGOs in round tables, works on national drug strategies). This trend is reflected in the performance of needle exchange programs, with the number of organisations operating NSPs rising from 10 in 2004 [100] to 29 in 2012 [100]—almost threefold increase. The number of clients increased by nearly 260% in 2004–2013 [100, 101] and the number of needles distributed in the country by over 680% [101, 102].

Significant changes of critical structural factors, especially political support, drug policy paradigm (including adopting a new drug strategy), financing framework and the amount of funds available, took place during 2011–2014. First, radical cuts in public expenditures in drug policy and especially harm reduction took place in 2011. The results were visible already a year after; the number of distributed needles has fallen by 35%, while the number of clients remained stable [103]. Although the situation started to recover in 2012–2013 slowly, the introduction of the new drug strategy followed by the politically motivated fight against certain services in Budapest resulted in closing down two country’s largest NSPs. Ever since, the indicators show poor performance, with the number of organisations providing services decreasing from 29 in 2014 [100] to 23 in 2017 [7], the number of needles distributed in the country falling by 70% (8101) and the number of clients decreasing by 50% (8101).

## Discussion

This study attempted to contribute to the scholarship on drug policy and harm reduction through addressing a relatively unexplored area, i.e. the environment where needle exchange programmes operate. Contrary to the majority of researches undertaken on structural barriers to HIV prevention among people who inject drugs, which are focused on an individual, this study focused on organisations providing services, putting them in the centre of attention. It answered three major questions: what the structural barriers and facilitators to needle exchange service delivery in Visegrad countries are, how they differ between analysed countries and how they impact the NSP service delivery.

The data sources can be considered a major limitation of this study. As mentioned in the “Methods” section, the qualitative data were primarily collected through interviews with employees of needle exchange programmes. As such, they are characterised by a certain level of subjectivity. This limitation was addressed by use of complementary sources, primarily relevant documents, legal acts, reports and press publications. One of the ways to develop a fuller picture of the analysed phenomenon could be extending the data collection to relevant policy-makers.

## Conclusions

This study identified 24 themes (structural barriers and facilitators) across 11 categories on three levels (culture, state, local). They include issues related to the broader society (e.g. morality), politics and policy on state and local level, frameworks and amounts of funding, the situation on the education labour market, and attitudes of local communities.

Based on the analysed data, it seems that structural barriers play a significant role when it comes to the performance of service delivery. Both cross-case and within-case analysis confirmed that the numerous and severe structural barriers are related to poor NSPs performance and the other way around, the presence of numerous facilitators is related to services’ development.

This study contributes to both theory and practice. First, it demonstrates that the ecological model can be successfully applied to study organisations. Second, it fills the gap in the research, identifying and classifying a set of structural factors in the environment of NSPs in the ECE region. It can thus serve as a starting point for further investigations involving other geographical or policy areas.

Regarding the practical relevance, the study demonstrated that structural factors identified are not independent; on the contrary, they are often intertwined and affect one another, creating a complex system of relationships. Therefore, in case of any desired changes, it is not sufficient to address them individually, one by one. The efforts aiming to shift the situation need to be multilevel, targeting numerous areas of barriers’ presence simultaneously, to the extent possible.

## Additional file

Additional file 1: The summarised version of the interview protocol. (DOCX 14 kb)

## Data Availability

The datasets generated and/or analysed during the current study are not publicly available due to constituting a source for the author’s ongoing PhD research but can be obtained from the corresponding author on reasonable request.

## Abbreviations

CSFD: Civil Society Forum on Drugs
ECE: East-Central Europe
EU: European Union
HIV: Human immunodeficiency virus
NSP: Needle and syringe exchange programme
OST: Opioid substitution treatment
PWUD: People who use drugs
WE: Western Europe

## Footnotes

1 The calculation was based on the data from 17 organisations where low-threshold NSPs are the only or main activity, or in cases of which it was possible to determine the budget attributable to the NSP service of the organisation.
