# Supplementary material for: Needle exchange programmes in Visegrad countries: a comparative case study of structural factors in effective service delivery
Source: Harm Reduct J. 2019 Sep 3;16:54. doi: 10.1186/s12954-019-0323-5 (PMC6724252; doi:10.1186/s12954-019-0323-5)
Supplement: Supplementary file 1 — The summarised version of the interview protocol. (DOCX 14 kb) [file 12954_2019_323_MOESM1_ESM.docx]

# Additional file 1

Annex 1. The summarised interview protocol.

1. Please introduce the organization

2. Can you tell me something more about the organization employees and volunteers?

3. Do you keep communication/supervision activities with your employees and volunteers to get feedback from them on various issues?

4. Where do you get your funding from and what is your yearly budget?

5. How does the organization work on everyday basis? What proportion of the time is spent on particular activities (including not mission related, e.g. administration?

6. What are the relationships of NGO with clients?

7. What are the relationships of NGO with donors and how does it influence the organisation’s work, if at all?

8. Do you experience any tensions between your organisation’s mission and is expected by the donors?

9. What are the relationships of NGO with law enforcement – police and how does it influence your work, if at all?

10. What are the relationships of NGO with local government and how does it influence your work, if at all?

11. What are the relationships of NGO with local community and how does it influence your work, if at all?

12. What are the relationships of NGO with other organisations working in the field and how does it influence your work, if at all?

13. What is the situation of drug policy in the country (approach towards drugs) and how does it influence your work, if at all?

14. Do you consider your work effective? Is there anything that could be improved? How?

15. How would your organisation’s situation look like in the ideal world?

16. Any other comments.
